# Genome-Wide Identification of *Brassica napus* PEN1-LIKE Genes and Their Expression Profiling in Insect-Susceptible and Resistant Cultivars

**DOI:** 10.3390/cimb44120435

**Published:** 2022-12-15

**Authors:** Lei Sheng, Zengbei Feng, Zhongping Hao, Shumin Hou

**Affiliations:** Crop Research Institute, Anhui Academy of Agricultural Sciences, Hefei 230031, China

**Keywords:** *Brassica napus*, insect resistance, homologous gene, bioinformatics analysis

## Abstract

Recently, it has been reported that a gene (PEN1) in *Arabidopsis thaliana* is highly resistant to *Plutella xylostella*. We screened all the homologous genes of PEN1 in *Arabidopsis thaliana* and found that the motif of these genes was very conserved. At present, few insect resistance genes have been identified and characterized in *Brassica napus*. Therefore, we screened all the homologous genes containing this motif in the *Brassica napus* genome and systematically analyzed the basic information, conserved domain, evolutionary relationship, chromosomal localization and expression analysis of these genes. In this study, 12 PEN1 homologous genes were identified in the *Brassica napus* genome, which is more than the number in *Arabidopsis thaliana*. These genes are unevenly distributed on the 12 chromosomes in *Brassica napus*. Furthermore, all the PEN1 homologous genes contained light responsiveness elements, and most of the genes contained gibberellin-responsive elements, meJA-responsive elements and abscisic-acid-responsive elements. The results will provide a theoretical basis for screening insect resistance genes from the genome of *Brassica napus* and analyzing the molecular mechanism of insect resistance in *Brassica napus*.

## 1. Introduction

*Brassica napus* is the main oil crop in the world and is the most important source of vegetable oil [1]. In addition, the seeds of *Brassica napus* are an important source of dietary protein for humans worldwide [2]. China has the largest cultivated area of *Brassica napus* in the world; the annual output of rapeseed was over 12 million tons, and rapeseed oil accounts for 40 percent of vegetable oil consumption [3]. At present, insect pests are one of the major problems affecting rapeseed yield [4]. Oilseed rape is susceptible to many fungal diseases and insect pests [5]. A global survey among 22 experts from 10 countries on major biotic constrains of oilseed rape production in 2019 revealed 37 insect pests that were present in oilseed rape or mustard since 2016 [6]. China’s oilseed rape production is divided into two major growing areas: winter and spring oilseed rape areas. Winter oilseed rape accounts for about 90%, and spring oilseed rape accounts for about 10% of total planting area in China. In recent years, winter oilseed rape areas have gradually expanded to the north of China. The main oilseed rape pest is aphid, and the geographic range and survival time have increased because of the amplification of winter oilseed rape [7]. It has been reported that aphid infects oilseed rape with the virus it carries, resulting in a 70 to 79% yield reduction [8].

The methods of pest control are mainly divided into chemical control, biological control, physical control agricultural control, and so on [4]. Among them, the starting point of agricultural control is the general concept of agro-ecosystems, its goal is to increase crop yield, improve farmland environment and create an environment that is suitable for crop growth and not conducive to the survival and growth of pests, so as to control the pest density under the condition that the economic loss permits [9]. Agricultural control is mainly achieved by adjusting farming systems and breeding insect-resistant varieties. Using insect-resistant varieties and strengthening plant defense systems is one of the most environmentally friendly and economical methods of insect control. Various defense traits of host plants affect the reproduction of herbivorous insects [10,11,12,13]. Phytochemicals may have repellent, deterrent, anti-nutritional and anti-digestive effects, thus interfering with the physiological functions of herbivores and reducing their development and survival rate [14,15,16,17,18]. For example, the distribution and concentration of glucosinolates and myrosinase in cruciferous plants have a significant effect on the population of their pests [19,20,21].

Insect-feeding-induced plant defense is regulated by a cascade of signals. Jasmonic acid (JA) and salicylic acid (SA) pathways induce a defense system against herbivores in cruciferous plants [22]. The interactions of these signaling pathways may be antagonistic or synergistic [23].

Synthetic JA has no direct toxicity or inhibitory effect on herbivores; however, it induces toxic chemicals (alkaloids, phenols and terpenes) in plant organs, thereby limiting attack by herbivores [24]. In addition, induced defense may enhance the plant’s constitutive defense against subsequent attackers [25]. The results of Sharma and Mouttet showed that induction of the plant defense response leads to the production of secondary metabolites, by which the first attack enhances the ability of the plant to resist the second attack [26,27].

At present, few rapeseed germplasm resources with insect resistance genes have been identified and characterized, and there are few studies on insect resistance genes in *Brassica napus* at home and abroad. In recent studies on the function of PEN1 gene in *Arabidopsis thaliana*, it was found that transgenic plants overexpressing PEN1 showed strong resistance to the infection of the Diamondback moth [28]. Therefore, we screened all the homologous genes of PEN1 in *Arabidopsis* and found that the motif of these genes was very conserved. Then, we screened all the homologous genes containing this motif in the *Brassica napus* genome and systematically analyzed the basic information, conserved domain, evolutionary relationship, chromosomal localization and expression analysis of these genes in order to predict the possible role of PEN1 homologous gene in the molecular mechanism of insect resistance in *Brassica napus*. The results of this study will provide a theoretical basis for screening insect resistance genes from the genome of *Brassica napus* and analyzing the molecular mechanism of insect resistance in *Brassica napus*.

## 2. Results

### 2.1. Identification, Characterization and Subcellular Localization of PEN1 Homologous Genes in Brassica napus

According to the retrieval results of two genome comparisons, we identified 12 PEN1 homologous genes in the *Brassica napus* genome (Table 1). The physiochemical characterizations of 12 PEN1 homologous genes were analyzed, and the results showed that the amino acid length ranged from 77 (*BnaA03g03820D*) to 445 aa (amino acids) (*BnaA04g10140D*); the molecular weight (Mw) ranges from 8.79 (*BnaA03g03820D*) to 49.87 KD (kilodalton) (*BnaA04g10140D*); the theoretical isoelectric point (pI) ranges from 4.61 (*BnaA04g10140D*) to 9.23 (*BnaC04g33950D*); the instability index ranged from 17.73 (*BnaA10g20460D*) to 53.78 (*BnaC06g01360D*); the aliphatic index ranged from 74.72 (*BnaA04g10140D*) to 125.32 (*BnaA03g03820D*); the grand average of hydropathicity (GRAVY) ranged from −0.431 (*BnaA04g10140D*) to 0.156 (*BnaA10g20460D*) (Table 1). In terms of physiochemical characterizations, the theoretical isoelectric points (pI) of most genes were less than 7.0, indicating weak acidity. Only the theoretical isoelectric points (pI) of *BnaC04g05920D* and *BnaC04g33950D* were more than 7.0, indicating weak alkalinity. Among the 12 PEN1 homologous genes, the instability indexes of 8 genes were all less than 40, indicating that the proteins of these genes were highly stable. The grand average of hydropathicity (GRAVY) of most genes was less than 0, indicating that they were all hydrophilic proteins. The result of subcellular localization showed that seven genes were located in the nucleus, and the other five genes were located in the cytoplasm.

### 2.2. Multiple Sequence Alignment and Phylogenetic Analysis

**Figure 1:**
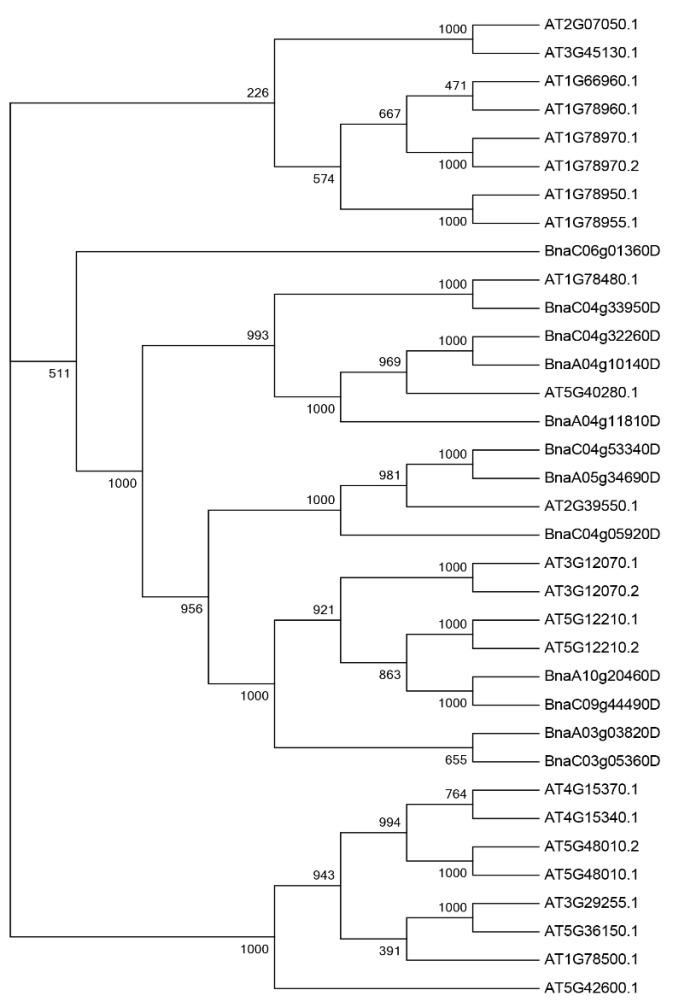
Phylogenetic analysis of proteins for PEN1 homologous genes from *Brassica napus* and *Arabidopsis thaliana*. Thirty-five protein sequences were used to construct the NJ tree with 1000 bootstraps.

In order to further understand the evolutionary relationship of PEN1 homologous genes, multiple sequence alignment was performed for the full-length protein sequences of 23 PEN1 homologous genes in *Arabidopsis thaliana* and 12 PEN1 homologous genes in *Brassica napus.* The phylogenetic tree was constructed by neighbor joining (NJ) in MGEA7.0 (Figure 1). It can be seen from the phylogenetic tree that all PEN1 homologous genes can be divided into three evolutionary branches, each containing 8, 19 and 8 members. Among them, the genes in branch 1 and branch 3 are both *Arabidopsis* genes, the remaining seven *Arabidopsis* genes and all of the genes in *Brassica napus* were in branch 2. The result indicated that 12 PEN1 homologous genes in *Brassica napus* were more closely related to the 7 *Arabidopsis* genes (Figure 1).

### 2.3. Conserved Motifs and Physical Locations

**Figure 2:**
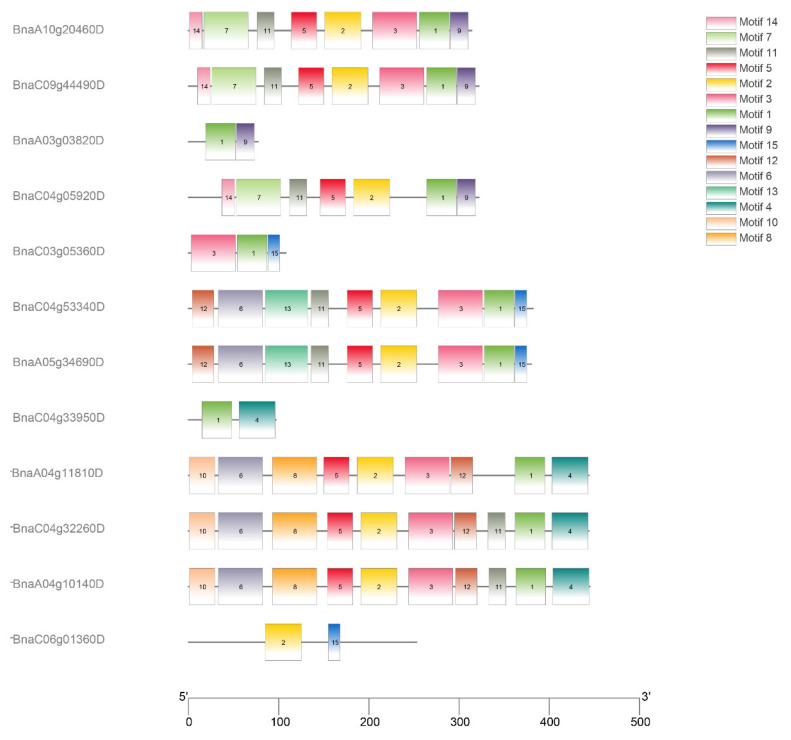
Distribution of conservative motifs in PEN1 homologous genes.

**Figure 3:**
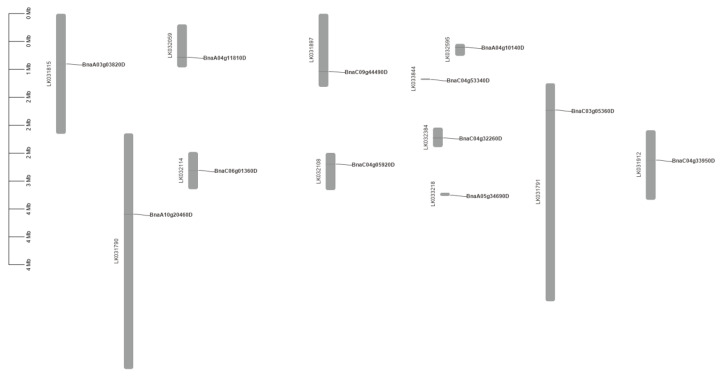
Distribution of PEN1 homologous genes on the 12 scaffolds in the *Brassica napus* genome.

The conservatism of protein sequences for PEN1 homologous genes in *Brassica napus* was analyzed using MEME. As shown in Figure 2, the predicted domains of *BnaA10g20460D*, *BnaC09g44490D* and *BnaC04g05920D* are exactly the same, except that *BnaC04g05920D* lacks motif 3, and the predicted domains of *BnaC04g32260D*, *BnaA04g10140D* and *BnaA04g11810D* are exactly the same, except that *BnaA04g11810D* lacks motif 11. The predicted domains of *BnaC04g53340D* and *BnaA05g34690D* are identical. All the above eight genes contain motif 1, motif 2 and motif 5. Among the remaining four genes, *BnaA03g03820D*, *BnaC04g33950D* and *BnaC06g01360D* only contain two domains, while *BnaC03g05360D* only contain three domains. All the 12 PEN1 homologous genes except *BnaC06g01360D* contain motif 1, indicating that motif 1 is a very conserved and important region in PEN1 homologous genes and may play a very important role in gene function or structure. Physical location showed that 12 PEN1 homologous genes were unevenly distributed on the 12 scaffolds in *Brassica napus* (Figure 3).

### 2.4. Gene Structure and Cis-Acting Element Analysis

**Figure 4:**
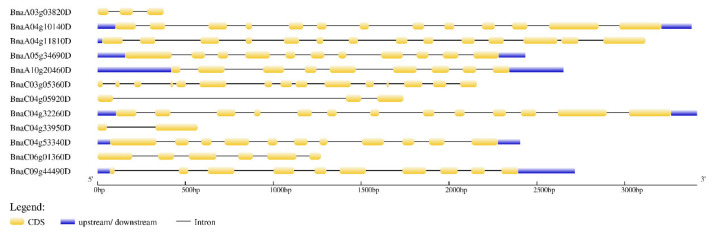
Exon-intron structures of PEN1 homologous genes. The lengths of the exons and introns of each PEN1 homologous gene are drawn to scale.

As can be seen from Figure 4, among the 12 PEN1 homologous genes, 8 genes contain 9–15 exons, and 4 genes contain 2–6 exons. The length and number of exons of *BnaA10g20460D* and *BnaC09g44490D* were similar, which was consistent with their motif composition. The same is true for *BnaC04g53340D* and *BnaA05g34690D*, *BnaC04g32260D* and *BnaA04g10140D*. Generally, the length and number of exons are similar between closely related genes, indicating that these genes are relatively conserved in evolution.

**Figure 5:**
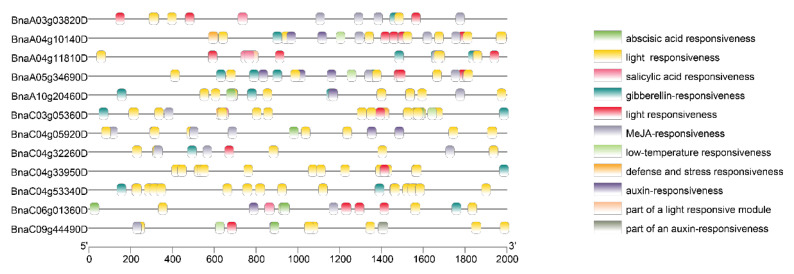
The prediction results of cis-acting element of PEN1 homologous genes.

By means of analysis of cis-acting elements in the promoter region, we identified multiple cis-acting elements related to hormone response and abiotic and biological stress (Figure 5). We found that all the PEN1 homologous genes contained light responsiveness elements, and most of the genes contained gibberellin-responsive elements, meJA-responsive elements and abscisic-acid-responsive elements. These results suggest that PEN1 homologous genes may regulate the growth and development of *Brassica napus* through different response pathways related to hormone response and abiotic and biological stress. In addition, only *BnaA04g10140D* and *BnaC03g05360D* contained defense and stress responsiveness elements among all PEN1 homologous genes, suggesting that these two genes may play important roles in response pathways related to defense and stress in *Brassica napus*.

### 2.5. Tissue Expression Analysis

**Figure 6:**
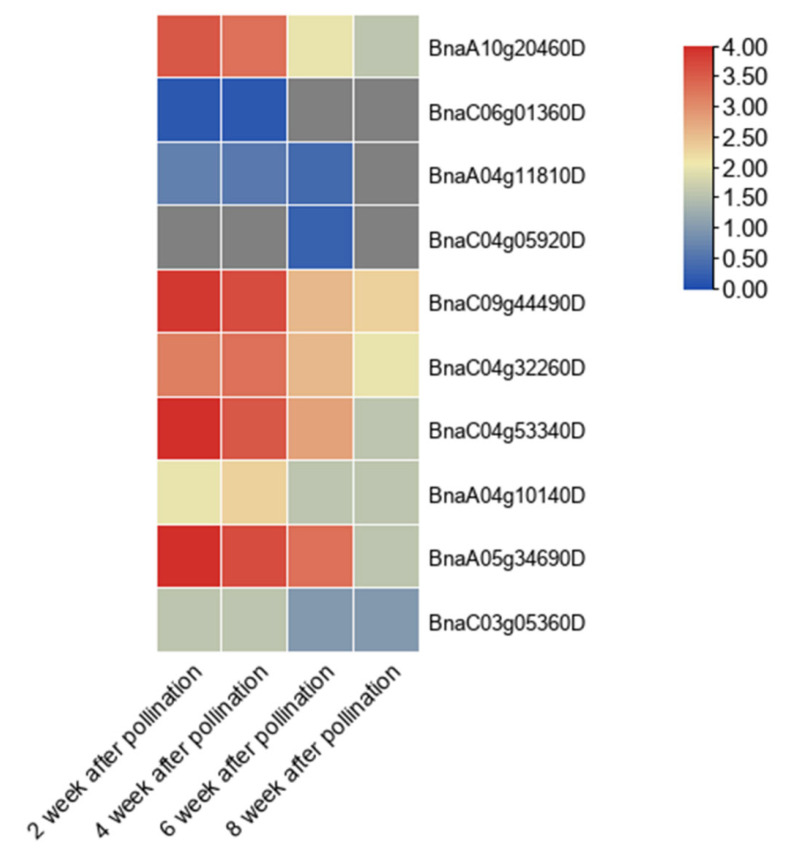
Expression profile of PEN1 homologous genes in different stages of *Brassica napus* seeds. The heat map was generated based on log_2_ transformed values of the ratio of FPKM in the treated groups to control groups. The redder color indicates higher expression of related genes.

According to the expression calorigram, among 12 PEN1 homologous genes, 5 genes (*BnaA10g20460D*, *BnaC09g44490D*, *BnaC04g53340D*, *BnaA05g34690D*, *BnaC04g32260D*) were highly expressed in *Brassica napus* seeds 2 to 4 weeks after pollination. The expression level gradually decreased in seeds 6 to 8 weeks after pollination. However, the expression of other genes was very low in seeds at different stages of development (Figure 6).

**Figure 7:**
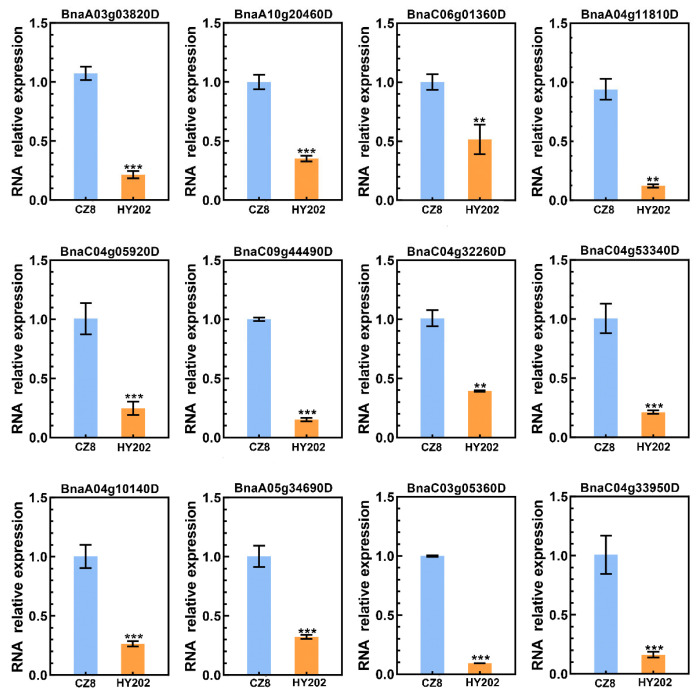
The expression patterns of PEN1 homologous genes in the leaves of two *Brassica napus* cultivars. Asterisks indicate significant differences (** *p* < 0.01, *** *p* < 0.001; *t*-test).

According to the results of fluorescence quantitative PCR, we found that there were significant differences in the expression of PEN1 homologous genes in the leaves of two *Brassica napus* cultivars (Chuangza 8 and Heyou 202). In this study, the aphid resistance levels of several *Brassica napus* varieties were identified using the method of indoor inoculation of insects and aphid index evaluation. Combining the two evaluation methods of accumulative aphid index and aphid situation index, Chuangza 8 and Heyou 202 were screened for fluorescence quantitative PCR after three repetitions (Table S2). Chuangza 8 is a highly resistant variety, and Heyou 202 is a highly susceptible variety. As shown in Figure 7, among the 12 PEN1 homologous genes, the expression of 9 genes in Chuangza 8 was significantly higher than that in Heyou 202. The result indicated that these nine genes may play an important role in the regulatory molecular mechanism of insect resistance in *Brassica napus*.

## 3. Discussion

The common biological stress in *Brassica napus* production seriously restricts the development of China’s *Brassica napus* industry. Therefore, improving the insect resistance of *Brassica napus* is of great significance for the development of China’s *Brassica napus* industry. At present, no monogenic resistance against any insect pest has been reported in oilseed rape, and no cultivars could show resistance against any of the commercially important insect pests [29]. Additionally, the primary germplasm pool of *Brassica napus* is thought to lack resistances to many insect pests [5]. Therefore, the research on insect resistance genes in *Brassica napus* remains to be explored at home and abroad.

*Brassica* plants contain many groups of secondary metabolites which are sometimes lineage specific. Secondary metabolites are often associated with regulation of growth, development and plant defense [30]. Major secondary metabolites associated with insects in the Brassicaceae include terpenoids, phytosterols, flavonoids, phenolics, cyanogenic compounds and alkaloids [31]. However, most of the studies were about the effects of glucosinolates [32,33]. The effects of other secondary metabolites in the Brassicaceae are scarce [34], which makes modifying secondary metabolites for insect resistance a breeding target with a medium- to long-term perspective [5].

Recent studies have shown that, when the *Arabidopsis* PEN1 gene (AT4G15340) was overexpressed, the plants showed strong resistance to *Plutella xylostella* infection [28]. Pentacyclic triterpene synthase 1 (PEN1) has been reported to be a key enzyme in the biosynthesis of the volatile homoterpene (3E)-4,8-dimethyl-1,3,7-nonatriene (DMNT) [35]. Additionally, DMNT repels *Plutella xylostella*, eventually killing larvae by disrupting their peritrophic matrix (PM) [28]. In general, sequences with high similarity retained during long-term evolution are conserved and usually have similar functions. In this study, 12 PEN1 homologous genes in *Brassica napus* were identified by bioinformatics methods and analyzed systematically from the aspects of phylogenetic evolution, gene structure, protein physicochemical properties, cis-acting elements and gene tissue expression characteristics. Through the analysis of cis-acting elements in the promoter region, we found that all the PEN1 homologous genes contained light responsiveness elements. Most of the genes contained a gibberellin-responsive element, meJA-responsive element and abscisic-acid-responsive element. Since plant hormones play an integral role in plant defense responses [36], among them, jasmonate (JA) and salicylic acid (SA) pathways induce defense systems against herbivores in cruciferous plants [37,38]. Therefore, we hypothesized that the PEN1 homologous genes might regulate the resistance response of *Brassica napus* to pests through different hormone response pathways. In addition, among all the PEN1 homologous genes, only *BnaA04g10140D* and *BnaC03g05360D* contained the defense and stress responsiveness element, which suggests that these two genes may play important roles in the defense and stress response pathways of *Brassica napus*. According to the results of fluorescence quantitative PCR, we found that the expression level of 9 PEN1 homologous genes in Chuangza 8 was significantly higher than that in Heyou 202, including these two genes. Based on the above analysis, we can focus on further functional verification of *BnaA04g10140D* and *BnaC03g05360D* in the future.

In recent years, transgenesis has been broadly applied to protect some major crop species against coleopteran and lepidopteran insect pests. Since the mid-1990s, genes for endotoxins have been used commercially in transgenic crops, such as maize and cotton, but not in oilseed rape [39]. The aim of this study was to provide a theoretical basis for screening insect resistance genes from the *Brassica napus* genome and analyzing the molecular mechanism of insect resistance in *Brassica napus*. Subsequent studies will focus on this direction to verify the function of genes and their participation in the molecular mechanism of insect resistance, in order to lay a foundation for the study of insect resistance in *Brassica napus*.

## 4. Materials and Methods

### 4.1. Data Sources

The *Arabidopsis thaliana* genome data was obtained from arabidopsis information repository (TAIR, https://www.arabidopsis.org/, accessed on 19 April 2022), and the *Brassica napus* genome data was obtained from the Ensembl database (http://plants.ensembl.org/index.html, accessed on 19 April 2022). Then, the protein sequences of *Arabidopsis* PEN1 homologous genes were searched and downloaded from PlantTFDB [40] (http://planttfdb.gao-lab.org/, accessed on 19 April 2022) as the probe sequence.

### 4.2. Identification and Physiochemical Characterization of PEN1 Homologous Genes in Brassica napus

The local genome databases of *Arabidopsis thaliana* and *Brassica napus* were constructed. The protein sequence of *Arabidopsis* PEN1(At4g15340) was used as the probe sequence, and the threshold E value was set as 10^−5^. The local BLAST program was used for comparative retrieval, and the protein sequences of candidate genes were obtained. The hidden Markov model (HMM) of PF00432 was downloaded from the Pfam database (http://pfam.Xfam.org/, accessed on 19 April 2022) [41]. Then, the downloaded HMM model was used as probe sequence to compare and retrieve the protein sequence data of *Brassica napus* using Hmmersearch, which is the subroutine in HMMER(V3.0) software [42], and the threshold E value was set to be less than 10^−5^. The existence of the Prenyltransferase domain of candidate genes was verified by online tools Conserved Domain Search (https://www.ncbi.nlm.nih.gov/Structure/cdd/wrps-b.cgi, accessed on 19 April 2022) and SMART database [43] (http://smart.embl-heidelberg.de/, accessed on 19 April 2022). Finally, PEN1 homologous genes in the *Brassica napus* genome were obtained by sorting out redundant and pseudogenes according to the retrieval results of two genome comparisons.

The subcellular locations of PEN1 homologous genes were predicted using WoLF PSORT (https://wolfpsort.hgc.jp/, accessed on 6 May 2022). The basic physicochemical properties were analyzed using protparam tools in ExPASy [44] (https://web.expasy.org/protparam/, accessed on 19 April 2022), which include the amino acid length, molecular weight, theoretical isoelectric point, grand average of hydropathicity, instability index, aliphatic index, and so on.

### 4.3. Multiple Sequence Alignment and Phylogenetic Analysis of PEN1 Homologous Genes in Arabidopsis thaliana and Brassica napus

The protein sequences of PEN1 homologous genes in *Arabidopsis thaliana* and *Brassica napus* were analyzed by DNAMAN software. Based on the multiple comparison results, the phylogenetic tree was constructed by MGEA 7.0 program [45], using the neighbor joining method with a bootstrap value of 1000, considering position correction and pairwise deletion.

### 4.4. Conserved Motifs and Chromosomal Locations of PEN1 Homologous Genes in Brassica napus

The conserved sequences and functional sites of the full-length protein sequence were analyzed using MEME (https://meme-suite.org/meme/tools/meme, accessed on 19 April 2022) [46] for PEN1 homologous genes. Fifteen motifs were set, and the rest of the parameters were default (Table 2). The above results were downloaded, and TBtools [47] was used to plot. The location of PEN1 homologous genes were obtained based on genome-wide data of *Brassica napus*, and the physical location of PEN1 homologous genes was mapped using TBtools.

### 4.5. Gene Structure and Promoter Element Analysis of PEN1 Homologous Genes in Brassica napus

The function of Gene Structure View in TBtools was used to visualize the gene structure of PEN1 homologous genes. Then, the sequences of PEN1 homologous genes and their upstream 1500 bp promoter sequences were extracted from the *Brassica napus* genome by TBtools. The cis-acting elements in the promoter region were predicted by Plant CARE (http://bioinformatics.psb.ugent.be/webtools/plantcare/html/, accessed on 19 April 2022) [48], and the number and distribution of cis-acting elements were shown using TBtools.

### 4.6. QRT-PCR Analysis

On the one hand, we downloaded the announced transcriptome data of different stages of b.napus rape seeds through an online website (https://www.ebi.ac.uk/gxa/experiments?species=Brassica+napus, accessed on 24 June 2022). Then, the expression data of PEN1 homologous genes were homogenized by log_2_, and the visualization of expression calorimetry was carried out using TBtools.

On the other hand, the experimental materials were the leaves of two *Brassica napus* cultivars (Chuangza 8 and Heyou 202) in the five-leaf stage. RNA was extracted from leaves, and the quality was evaluated by electrophoresis strips and spectrophotometric measurement [49]. The cDNA was synthesized using HiScript III RT SuperMix (Vazyme). RT-qPCR was performed on the Thermo Scientific PikoReal 96 RT-PCR instrument. In RT-qPCR, each reaction had a total volume of 20 µL, consisting of 2 µL diluted cDNA, 10 µL of AceQ qPCR SYBR Green Master Mix (Vazyme), 1 µL forward and reverse primers, and 6 µL RNA-free water. The qPCR program was performed under the following conditions: 95 °C 5 min, followed by 40 cycles of 95 °C for 10 s, 60 °C for 30 s. The Bntubulin gene was used as an internal control, and the 2^−ΔΔCt^ method was employed to calculate the relative expression of genes [50,51]. The primers used for RT-qPCR are listed in Supplementary Table S1. All the experiments were conducted with three biological and three technical replicates.

## Figures and Tables

**Table 1:** Basic information of PEN1 homologous genes in *Brassica napus*.

Gene ID	pI	Mw (KDa)	Amino Acids (aa)	Instability Index	GRAVY	Aliphatic Index	Subcellular Localization
BnaA03g03820D	5.12	8.79	77	27.59	0.083	125.32	nucleus
BnaA10g20460D	5.06	34.52	314	17.73	0.156	104.55	cytoplasm
BnaC06g01360D	5.85	29.40	253	53.78	−0.176	87.08	cytoplasm
BnaA04g11810D	4.65	49.76	444	37.33	−0.289	87.18	nucleus
BnaC04g05920D	9.01	11.91	108	27.37	0.150	103.98	nucleus
BnaC09g44490D	5.06	35.38	322	22.36	0.136	100.75	cytoplasm
BnaC04g32260D	4.66	49.76	444	39.90	−0.407	78.20	nucleus
BnaC04g53340D	4.64	42.17	382	50.22	−0.256	80.24	nucleus
BnaA04g10140D	4.61	49.87	445	41.97	−0.431	74.72	nucleus
BnaA05g34690D	4.65	41.94	380	51.60	−0.230	81.95	nucleus
BnaC03g05360D	5.80	36.19	322	23.43	−0.024	92.02	cytoplasm
BnaC04g33950D	9.23	11.21	97	27.47	−0.248	82.37	cytoplasm

**Table 2:** Motif information of PEN1 homologous genes.

Number	Sequence	Length
1	LCSQVENGGFSDKPGDPRDIYHTYYGLSGLSLLE	34
2	TEKAGDYILSCQTYDGGFGGEPGSESHGGQTYCGVATLAJI	41
3	NWIVHRQGVEGGFQGRTNKLVDGCYTFWQAAPLVLJQRVYSIDKLALHGF	50
4	DTPPLTRDILGGYANHLEPVHLLHNVVMDRYNEAIEFFHRA	41
5	QDEDGGFSGHTMGEVDVRFSYIAISIASI	29
6	IATQPFSVEIQRDKQLDYLMNGLRQLGPSFSSLDANRPWVCYWIIHSIAL	50
7	EKKKESFESVVMDHLRMNGAYWGLTTLDLLDKLGSVSVDEVVSWLMTCQH	50
8	NNAIDFLGRCQGSDGGYGGGPGQLPHLATSYAAVNTLVTLGGEKAFSSIN	50
9	YPGVKAIDPAYALPVDVINRI	21
10	MEELPSITVSQREQFLVENDVFGMYSYFD	29
11	TGHDPHLASTYIALRILPVF	20
12	HMSQGEDEDHEEHAHDEDDPEDSDE	25
13	ATDRVDKDVVAKWVLSFQAFPSNRALLKEGEFYGFYGSRSSQFPIDEN	48
14	MGQLVADKHVRYILM	15
15	EPGLSPLCPELGLP	14

## Data Availability

All the data relevant to the study are included in the article or uploaded as Supplementary Materials.

## Supplementary Materials

The following supporting information can be downloaded at: https://www.mdpi.com/article/10.3390/cimb44120435/s1. Table S1: Real-time fluorescence quantitative PCR primers; Table S2. The class of resistant levels of different *Brassica napus* varieties to aphids.
