# Supplementary material for: Genome-Wide Identification of Brassica napus PEN1-LIKE Genes and Their Expression Profiling in Insect-Susceptible and Resistant Cultivars
Source: Curr Issues Mol Biol. 2022 Dec 15;44(12):6385–96. doi: 10.3390/cimb44120435 (PMC9777220; doi:10.3390/cimb44120435)
Supplement: Supplementary file 1 [file cimb-44-00435-s001.zip › Supplementary Table S1.docx]

| Gene | Forward primer sequences | Reverse primer sequences |
| --- | --- | --- |
| BnaA03g03820D | AGGATTTGGAGAATGGAGGCA | TCAACAAGCAAAGCGTAGGC |
| BnaA10g20460D | CCGAGAAGCTCCCTGATGTT | CATCGTCCGGTCTGTCTGAG |
| BnaC06g01360D | GCGTATTCTCGGAGTGGGT | GGAGGCATCGGATTGGTT |
| BnaA04g11810D | TGTTTGAAGATTGTAGGAGGGT | CCAAGCGTGTTGAGCCAC |
| BnaC04g05920D | ATGGACATCAGAGTCGGGAC | CAGATCCGGTAAAGCTCCAG |
| BnaC09g44490D | CTTCTTCCTTCCAGATGGGT | GTTGTCAGTCCCCAGTATGC |
| BnaC04g32260D | TGGTGGATATGGTGGTGGTC | CGAAATGGCAGTGTAGCAGG |
| BnaC04g53340D | CGCACCATTATCAATCCCAG | CCCACTTCGCAACTACATCT |
| BnaA04g10140D | GCAATCGACTTTCTTGGACG | AGCACGCTCGCACATCTATT |
| BnaA05g34690D | GACGAAGATTCCGATGACTC | CGGAAGCAACTCGTACATCA |
| BnaC03g05360D | TCTTGGTGGGTGTTGTCGAG | ACAGCATCTTCTGGTCGGTC |
| BnaC04g33950D | TGTTACTGTCTAAGCGTTCT | TTGTTGTACCGATCCATG |

Table S1. Primers used for Real-time quantitative PCR.
