# Supplementary material for: Genome-Wide Identification of Brassica napus PEN1-LIKE Genes and Their Expression Profiling in Insect-Susceptible and Resistant Cultivars
Source: Curr Issues Mol Biol. 2022 Dec 15;44(12):6385–96. doi: 10.3390/cimb44120435 (PMC9777220; doi:10.3390/cimb44120435)
Supplement: Supplementary file 1 [file cimb-44-00435-s001.zip › Table S2.docx]

Table S2. The class of resistant levels of different *brassica napus* varieties to aphids.

| Variety | Accumulative aphid index | Class of resistant levels | Aphid situation index | Class of resistant levels |
| --- | --- | --- | --- | --- |
| Zhongyouza 39 | 8.42 | susceptible | 1.33 | highly susceptible |
| Qinyou 10 | 5.20 | resistant | 0.67 | resistant |
| Dehuiyou 88 | 6.69 | resistant | 0.91 | susceptible |
| Qinyou 28 | 6.64 | resistant | 0.81 | susceptible |
| Zheyouza 1403 | 7.06 | susceptible | 1.05 | susceptible |
| Heyou 202 | 10.90 | highly susceptible | 1.68 | highly susceptible |
| Zhongheza 418 | 6.36 | resistant | 0.97 | susceptible |
| Chuangza 8 | 4.73 | highly resistant | 0.58 | resistant |
